# Tailoring the Models of Transcription

**DOI:** 10.3390/ijms14047583

**Published:** 2013-04-08

**Authors:** Alena Pance

**Affiliations:** The Welcome Trust Sanger Institute, Genome Campus Hinxton, Cambridge CB10 1SA, UK; E-Mail: ap9@sanger.ac.uk; Tel.: +44-1223-834-244 (ext. 8710)

**Keywords:** transcription, promoter, reporter gene, transcription factor, chimera

## Abstract

Molecular biology is a rapidly evolving field that has led to the development of increasingly sophisticated technologies to improve our capacity to study cellular processes in much finer detail. Transcription is the first step in protein expression and the major point of regulation of the components that determine the characteristics, fate and functions of cells. The study of transcriptional regulation has been greatly facilitated by the development of reporter genes and transcription factor expression vectors, which have become versatile tools for manipulating promoters, as well as transcription factors in order to examine their function. The understanding of promoter complexity and transcription factor structure offers an insight into the mechanisms of transcriptional control and their impact on cell behaviour. This review focuses on some of the many applications of molecular cut-and-paste tools for the manipulation of promoters and transcription factors leading to the understanding of crucial aspects of transcriptional regulation.

## 1. Introduction

Proteins are the building blocks of cells, as integral parts of the cell structure and effectors of its functions. Though translation of the mRNA message, as well as modification and correct folding of the polypeptide chains are essential for the protein composition of a cell, expression of all the components begins by transcription from DNA. Thus, synthesis of mRNA is the first step that controls eukaryotic gene expression. Transcription consists of three main phases: initiation, when RNA polymerase II (Pol II) is recruited to the promoter and begins RNA synthesis; elongation, during which the transcripts are completed and termination, when Pol II and the full length mRNA disengage from the DNA template [1]. The process begins with the assembly of general transcription factors around the transcription start site (TSS) to form the pre-initiation complex (PIC), which is completed with the recruitment of Pol II. The dominant view of transcriptional regulation has been that this is the rate-limiting step of transcription and, therefore, the main point of regulation. More recent studies have shown that large sets of genes are also controlled at the step of elongation and Pol II processivity [2,3].

The mechanisms of transcriptional regulation are tailored to individual genes. Constitutive genes are mainly house-keeping proteins that form each cell and confer its basic functions. Transcription of these genes is constant, at a pace depending on the need and half-life of each protein. Regulated genes are proteins expressed in response to signals from the cell and its environment. These signals are transmitted to the nucleus in the form of active transcription factors that trigger transcription by binding to response elements mainly in the 5′ sequences or promoters of genes, activating the transcriptional machinery. A wide range of genes are expressed on demand, controlled by different mechanisms according to their function. Thus immediate early genes (IEG) are rapidly and transiently induced by modulation of the chromatin structure and PIC pre-assembly, with elongation being the main point of regulation [4,5]. On the other hand, late response genes (LRG) show a delayed activation, often mediated by IEGs, through a variety of mechanisms with control points at every step of transcription.

A wide variety of highly sophisticated technologies have been developed to study molecular events in more detail. However, in this review, we will concentrate on those using molecular cut-and-paste as a strategy to understand the mechanisms that drive and regulate transcription.

## 2. Regulatory Sequences: Promoters

### 2.1. Pasting Reporter Genes

The transcriptional regulation of a particular gene has been traditionally studied by analysing the abundance of the messenger RNA by Northern blot, RNA protection RT-PCR or quantitative RT-PCR [6]. Changes in transcript abundance can be correlated with extracellular stimuli, and the signalling pathways involved can be identified through inhibition or activation with chemicals. Genome-wide techniques, such as microarrays [7], whole genome tiling arrays [8] and RNA sequencing [9], make it possible to examine all the genes transcribed in a cell under specific conditions. Though extraordinarily powerful for the assessment of overall gene expression, these approaches do not give an insight into the molecular mechanisms involved in the regulation process.

**Figure 1:**
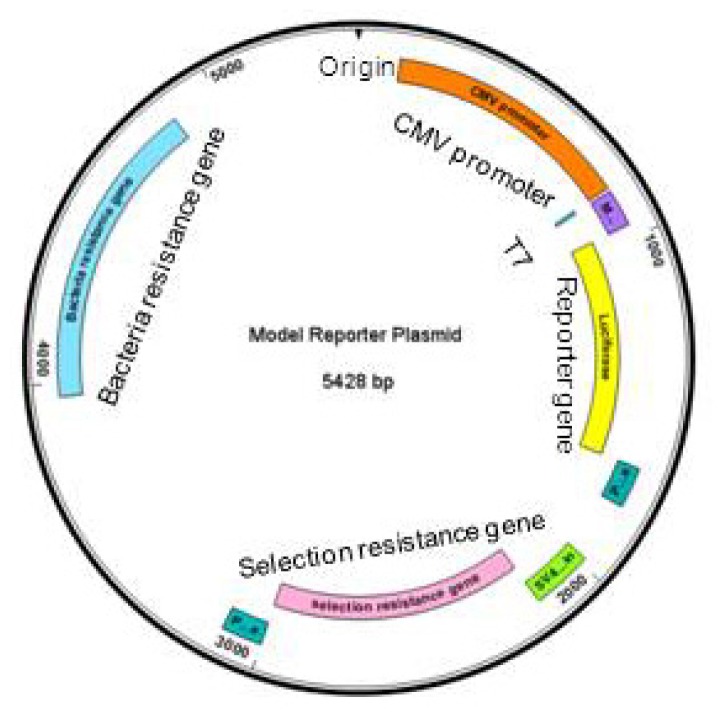
Basic elements of a generic reporter gene plasmid. Clockwise from the origin: CMV promoter (orange), T7, multiple cloning site (purple), reporter gene (yellow), poly adenylation sites (dark green), SV40 promoter and origin (light green), selection resistance gene (pink), poly adenylation site (dark green), bacteria resistance gene (blue).

The level of detail that could be studied increased considerably with the development of reporter genes. These consist of the 5′-flanking region or promoter of the gene of interest pasted to a gene, which becomes an indicator of the promoter’s activity (Figure 1). The recombinant construct in an expression vector is then transfected into cells and after the appropriate time for expression, the reporter gene is measured. After transfection, the cells can also be exposed to the desired stimuli to assess their effect on gene expression. The reporter gene can be detected directly by assaying for its mRNA or protein, but these methods are generally long and may not always be sufficiently sensitive and quantitative. Thus, the most popular reporter genes have a measurable activity that can be detected swiftly in easily performed quantitative assays of high sensitivity [10].

The bacterial enzyme, chloramphenicol acetyltransferase (CAT), catalyses the transfer of the acetyl group from acetyl-CoA to the substrate chloramphenicol. CAT has been widely used in recombinant constructs, because it is relatively stable in mammalian cells and has no eukaryotic equivalent that could interfere with the assay, so the activity measured corresponds exclusively to the construct introduced in the cells and reflects the activity of the promoter under study. The neuropeptide Y (NPY) promoter was characterised pasting 700 bp of the 5′-flanking sequence into a CAT reporter. It was shown that vasoactive intestinal peptide (VIP) induces the NPY promoter, and specific inhibitors attributed this activity to the cAMP-dependent protein kinase A (PKA) [11]. On the other hand, nerve growth factor (NGF) was shown to induce NPY mainly through activation of protein kinase C (PKC) [12]. Importantly, this molecular tool also permitted an insight into the cell-type specificity of gene expression, showing that introduction of constructs containing 5′-flanking regions of insulin coupled to CAT elicited preferential CAT activity when transfected into insulin-producing cells, while the activity of the chymotrypsin promoter was higher in chymotrypsin-producing cells. This demonstrated that promoters are responsive to stimulation and that particular cell types are equipped with the necessary machinery to activate the promoters of the proteins these cells need to express [13].

The reporter strategy has also provided the possibility of studying gene expression in complex organisms, such as the malaria parasite, *Plasmodium falciparum*. Its AT-rich genome and extensive repeats make the identification of regulatory sequences very difficult. Using a CAT reporter construct for transient expression in the parasite, a 5′ region of a gene called maebl was identified as harbouring the regulatory activity for the expression of this gene [14].

However, measuring CAT activity is a lengthy and laborious procedure that involves incubation of extracts from transfected cells with C^14^-labelled chloramphenicol, analysing the products by thin layer chromatography and quantifying the amount of acetylated chloramphenicol by scintillation counting or by exposure to X-ray films and densitometry. Additionally, the level of chloramphenicol conversion is highly dependent on the strength of the promoter, making analysis of weak promoters difficult [15]. These limitations can be circumvented by quantifying the protein directly using and enzyme-linked immunosorbent assay (ELISA), which maintains a sensitive range of detection.

Other commonly-used reporter genes include β-galactosidase, growth hormone (GH), β-glucuronidase (GUS), alkaline phosphatase (AP), green fluorescent protein (GFP), *etc*. In particular β-galactosidase, encoded by the *Lac Z* gene from *E. coli*, has been very useful for determining or normalising transfection efficiency. This enzyme catalyses the hydrolysis of β-galactoside sugars, such as lactose, and its activity can be assayed with a variety of substrates detected with a spectrophotometer, fluorometer, luminometer or *in situ* with histochemical staining. Enzymatic activity can also generate colour compounds that can be directly visualised on the microscope, such as the blue colour generated by the cleavage of X-gal, allowing direct visualisation of transfected cells. However, some mammalian cells have endogenous lysosomal β-galactosidase activity, so using higher pH of 7–8, pre-heating extracts to 50 °C or using negative controls becomes an important precaution [10].

In the 1980s, the luciferase gene from the firefly *Photinus pyralis* was cloned [16] and shown to be a highly sensitive reporter when transfected into mammalian cells [17]. In the presence of ATP, luciferase converts luciferin into oxyluciferin with concomitant emission of yellow-green light that can be quantified in a luminometer. The shorter half-life of luciferase as compared to CAT, for example, makes it particularly suitable for transient assays designed to assess inducible and short-lived events. Another advantage is the high activity of luciferase, which allows earlier detection of weak promoters in fewer cells, making the assay less dependent on high transfection efficiency [15]. Furthermore, luciferase activity can be normalised for transfection efficiency by co-transfecting a control construct in which a viral promoter controls expression of the sea pansy (*Renilla reniformis*) luciferase. *Renilla* has a different substrate requirement from the firefly luciferase, but its emission can also be detected by a luminometer in a dual assay that provides a reproducible, accurate and sensitive method to measure transcriptional activity in a simple and rapid detection assay.

The luciferase reporter gene has been used in a wide variety of studies examining complex interactions of transcription factors and molecules on promoters and their role in transcriptional regulation, sometimes providing links between systems and processes. For example, while studying the transcriptional regulation of the inducible nitric oxide synthase (iNOS or NOSII), we showed an interaction between the hormonal and immune systems. iNOS is an important component of inflammation and innate immunity, fulfilling a role of non-specific anti-microbial defence by secreting high levels of nitric oxide (NO), which quickly kills invading organisms. The toxicity of high levels of NO implies that the enzyme must be tightly regulated starting by transcription, which is only induced in response to stimulation by immune cytokines, bacterial compounds, *etc*. In a breast cancer cell line expressing the progesterone receptor (PgR+), progesterone (Pg) was found to increase cytokine activation of iNOS, while it has not effect in PgR− cells. The additive effect of Pg results in an increase of cell death, which might have implications for breast cancer biology [18].

Sometimes, determining regulatory sequences and crucial marks, such as the TSS, are not straight forward, as was the case of the human caspase family of proteins. Caspases are cysteine proteases that play an important role in cell death by apoptosis. Their promoters still remain largely uncharacterised, which was particularly intriguing in the case of caspase-2, because two isoforms with opposing effects on apoptosis had been described. A long isoform (casp-2L) was shown to induce programmed cell death, whereas the short isoform (casp-2S) could suppress it. In order to understand how expression of these isoforms is regulated, the 5′-flanking region of the casp-2 gene was cloned, and 100 bp fragments encompassing potential TSSs were introduced into a luciferase construct. This vector contains a viral SV40 enhancer, but requires insertion of a TSS to drive luciferase expression. It was shown that casp-2L uses a stronger TSS that determines the inclusion of a different exon 1 from casp-2S. In turn, the short isoform has a weaker TSS leading to an exon 1 that is skipped in casp-2L and causes a frame-shift that generates an early stop codon explaining the shorter protein. Putative promoters of the two isoforms were analysed in luciferase reporters, showing that casp-2L has a much stronger promoter than casp-2S, which correlates to the higher levels of the long isoform in cells [19]. The individual modulation of the casp-2S promoter by specific transcription factors without affecting casp-2L expression confirmed the functionality of both promoters [20,21]. Thus, the use of alternative promoters, as well as alternative promoter splicing became apparent as a mechanism for distinct translation initiation site usage regulating isoform expression.

### 2.2. Deletions and Mutations

**Figure 2:**
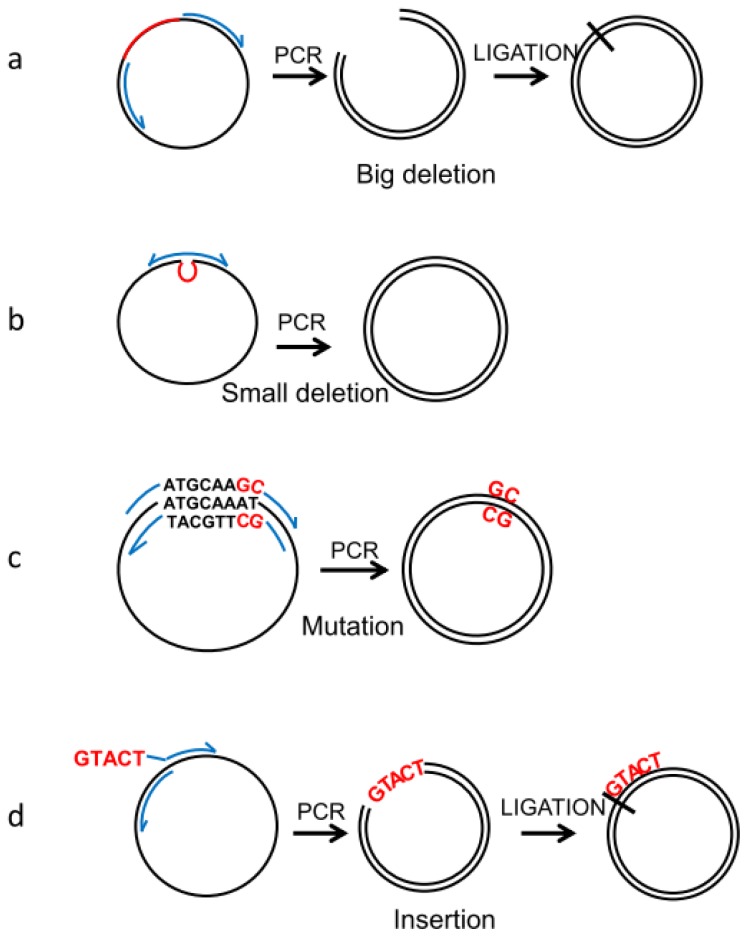
PCR-based manipulation of reporter genes. Modified regions are indicated in red, primers in blue and the plasmids in black. (**a**) Major regions are deleted by placing the primers outside of the region to be deleted to obtain a linear sequence that is ligated into a circular plasmid. (**b**) Primers are designed encompassing the flanking sequences of the small region to be deleted, which will be looped out in the resulting plasmid. (**c**) A few nucleotides are exchanged by placing the modified bases within the primers to be included in the synthesised plasmid. (**d**) Sequences can be inserted by placing them at the end of the forward primer and ligating into a circular plasmid.

Recombinant reporter vectors can be manipulated in order to characterise the precise role of un-translated regions (UTR) in the transcription of the gene they regulate. Particularly large promoters, such as the human iNOS, can be studied using this strategy. Up to 16 kb of the iNOS promoter have been analysed, inserting various 5′-flanking sequences of the gene into a luciferase reporter vector [22,23]. Several regions scattered throughout the promoter were defined that harbour cytokine-responsive elements, while large sections, such as the first 3.8 kb of the 5′-flanking sequence, do not seem to play a role in transcriptional activation, at least in response to cytokines [22]. Once large promoters were cloned into luciferase vectors, chunks could be deleted, either by enzymatic digestion or by high fidelity PCR (Figure 2a), to probe for active and inert regulatory regions, as was confirmed for the human iNOS [24]. This strategy not only confirmed and narrowed the location of crucial regulatory elements, but in some cases, deletions caused an increase of transcription, revealing the presence of repressors, as well [25].

Regulatory sequences are also be found in the 3′UTRs of genes. These are mainly implicated in the control of translation and mRNA stability, but in some cases, have been shown to play a role in transcription as well. 3′UTRs can be examined by insertion downstream of reporter genes, as was done for the mixed lineage leukaemia (MLL) proto-oncogene. Rearrangements of MLL with different partner genes involving the 3′UTR are associated with the development of acute leukaemia. Cloning the MLL 3′UTR downstream of luciferase showed strong repression of the gene, and a deletion analysis localised this effect to a 900 bp region of the proximal 3′UTR. The decrease in luciferase activity was paralleled by a marked reduction of mRNA, yet its stability was not compromised, suggesting a transcriptional mechanism. Insertion of a polyadenylation signal upstream of the repressive region restored mRNA synthesis, demonstrating that in order to repress, the 3′UTR has to be part of the transcribed unit. It was discovered that the repressive region of the MLL 3′UTR has the capacity to retain Pol II slowing the rate of transcription. The fusion with partner genes abrogates this effect leading to MLL overexpression and leukaemogenesis [26].

Identifying regulatory regions in promoters advanced the understanding of transcriptional regulation, but the role of transcription factors and their response to signalling pathways still remained obscure. Once a promoter-region was identified as bearing regulatory activity, its sequence can be examined for consensus transcription factor-binding sites that can be verified *in vitro* by electrophoretic mobility shift assay (EMSA) that also allows identification of the binding transcription factors with specific antibodies [27]. Once consensus transcription factor binding sites have been found in a promoter, their function can be directly assessed in reporter genes. Deleting or mutating consensus elements within promoters coupled to a reporter gene became the classical strategy of promoter analysis. These modifications are generally performed using high-fidelity PCR to amplify the whole reporter plasmid from primers containing the desired modifications (Figure 2). The primers contain the flanking sequence on either side of the binding element, so that it is excluded during amplification (Figure 2b), or a few nucleotide changes that will prevent the transcription factor from binding (Figure 2c). Mutagenesis led to the identification of crucial transcription factors for the activation of iNOS transcription in response to a variety of stimuli, including NFκB [23], AP-1 [28], STAT-1 [24] and Oct-1 [29,30]. The responsiveness of specific transcription sites to signalling pathways can also be examined by genetic manipulation. Using a site-specific deletion analysis in a luciferase reporter, we showed that AP-2 sites in the NPY promoter mediate cyclic AMP activation through PKA (unpublished data), while an AP-1 element is responsible for NGF stimulation via PKC [31]. With the same strategy, it was shown that the NPY receptor (NPY-Y1) is regulated by the same stimuli and its promoter contains elements responsible for this activation, demonstrating the coordination of gene expression [32].

The function of transcription factors can be studied directly and independently of the genes they regulate with the construction of transcription factor-specific reporter genes. In this approach, several copies of a binding element are placed in tandem upstream of a luciferase gene and its core promoter. Thus, four tandem copies of the κB enhancer that binds NFκB showed that NFκB-driven gene expression is dependent on p38 MAP kinase, which is partly due to activation of the TATA binding protein (TBP) [33]. We used the same reporter vector to demonstrate that NFκB activation is different in various sub-clones of a murine mammary cancer cell line, and this has repercussions on the gene expression and aggressivity of these tumour clones [34].

Transcription factor-specific reporters have also proven invaluable to refine DNA binding sites by determining the base requirements for protein interaction. This approach is particularly useful for the analysis of long binding sites, such as the 21 bases long RE1. The Neurone Restricted Silencing Factor (NRSF, REST) that binds RE1 is a transcriptional repressor that silences neuronal genes in non-neuronal cells. A search of the data base for genes containing RE1 revealed a certain variability in the base composition of the core consensus sequence, so in order to define the crucial bases for REST binding and function, different RE1 sequences were introduced in a CAT reporter. Repressive activity was assayed and matched with REST binding, identifying eight variable nucleotides that are not critical for REST binding, while the other 13 are more conserved [35]. The role and characteristics of transcription factor-binding sites became clearer with the use of reporter genes.

### 2.3. Insertions

The number of consensus elements in promoters and the complexity of transcriptional activation were clear indications that transcription factors generally do not act alone. They cooperate to induce activation, amplify or repress other protein’s signals, recruit other factors or co-factors to the DNA, interact with the basal machinery, and some of them can bend or modify the DNA structure. Some of these interactions occur through direct protein-protein binding, suggesting that the distance between binding sites might be important. In order to study these effects, Bertolino and Singh constructed a reporter vector containing an octamer site (Oct or POU) together with a core promoter and distal enhancer regulating the β-globin gene, whose RNA was measured directly to evaluate transcriptional activation [36]. They were able to show that by placing the Oct element close to the TATA box, the binding of Oct-1 could mediate distal enhancer activation. Increasing the distance between the POU element and the TATA box by a half (form 10 bp to 15 bp) or full (from 10 to 20 bp) helical turn of DNA, showed that POU activity was not affected by the positioning on the DNA, but a significant loss was observed with increased spacing. So, to mediate distal enhancer activation, the Oct site needs to be in close proximity to the core promoter owing to a direct interaction between Oct-1 and the TATA binding protein, TBP. We confirmed these observations in the context of iNOS transcription, by inserting small inert DNA sequences into a luciferase reporter of the iNOS promoter, to increase the separation between the TATA box and the Oct site by half a turn of DNA (5 bp) at a time (Figure 2d). Any increase disrupted transcriptional activity, indicating that the proximity between TBP and Oct-1 is important for PIC formation [30]. We went on to suggest that this mechanism allows recruitment of Pol II and initiation of transcription, so that the rate limiting-step is elongation of the transcripts rather than recruitment of Pol II [3].

Such studies began unravelling the complexity of promoter structure, locating regulatory elements as far as 100 kbp from the TSS and leading to the concept of enhancers. These are DNA sequences containing transcription factor binding sites, sometimes in clusters that regulate the activity of core promoters. It is the core promoters surrounding the TSS that serve as the docking site for the basal transcription machinery, ultimately leading to the assembly of the PIC with binding of Pol II [37,38]. The interaction between enhancers and promoters remains enigmatic, but it is thought that the transcription factors binding to enhancers are brought into proximity with the core promoter by chromatin loops, and the DNA flexibility is itself facilitated by the binding of certain transcription factors [39]. The spatial interactions between transcriptionally active regions of chromosomes can be studied directly by chromosome conformation capture (3C) and related techniques [40,41]. The composition of the core promoter is quite heterogeneous, and some of the factors binding in close proximity have a broad range of functions, including the interaction with distal enhancers and transmitting these signals to the transcription machinery.

## 3. Regulatory Genes: Transcription Factors

### 3.1. Modifications

The difficulty of assessing the contribution of particular transcription factors to transcriptional activation of a gene is complicated even further by the existence of transcription factor families with the same DNA-binding specificity. For example, Oct binding sites are recognised by several members of the POU family, such as Oct-1, Oct-2 and Pit-1; Sp1 binding sites are bound by Sp-1, Sp-2 and Sp-3 and the GATA motif by GATA-1, GATA-2, GATA-3 and GATA-6 [42]. An ingenious approach was devised to analyse the regulatory activities of the individual members of a family of transcription factors in an appropriate cell type. This strategy involved the mutation of DNA elements in reporter constructs to impair binding of wild-type proteins. Thus, a variant of the Oct consensus element (ATGCAAAT) was generated (A7G: ATGCAAGT) that is not recognised by the endogenous Oct-1 and Oct-2 proteins. A set of altered specificity transcription factors was then engineered by changing the specific amino acids that allow the mutant protein to bind the modified DNA element [42]. This method demonstrated that the Oct-1 and Oct-2 transcription factors are functionally redundant in regulating immunoglobulin (Ig) promoters in B-cells. Moreover, this redundancy is independent of the cell type and gene under study and lies in the DNA-binding POU domain of these proteins. If the Oct element is placed close to the core promoter, both Oct-1 and Oct-2 are capable of inducing transcription. However, when activation is dependent on a distal enhancer, differences between these factors emerge owing to the activation domains on either side of the POU domain, that confer specific activation capacity. As a consequence, immune-specific proteins, such as the immunoglobulins, are not expressed in non-immune cells, despite the presence of an active Oct-1.

### 3.2. Dominant Negative

A widely used method to understand the role of a protein is to inhibit either its activity by chemicals or its expression by siRNA and study the effect of this inhibition. It is also possible to use molecular tools to modify transcription factors in such a way that they lose their transcriptional activity. The transcription factor, REST, mentioned earlier, is composed of a zinc finger DNA-binding domain with affinity for the RE1 element and *N*- and *C*- terminal domains that recruit chromatin-modifying enzymes, including Histone de-acetylases (HDAC). So, by binding to its DNA element, REST causes tightening of the DNA nucleosome structure, making it inaccessible to the transcription machinery [43]. A modified version of REST was constructed in which the *N*- and *C*- terminal domains were deleted, while preserving the DNA binding domain. To facilitate detection, a c-myc-epitope tag was incorporated at the *C*-terminal, as well as GFP as a marker for transfection efficiency. This construct acts in a dominant-negative (DN) capacity to compete-out endogenous REST binding to RE1 [44]. Adenovirus constructs of REST and DN-REST were used for a genome-wide assessment of REST function using chromatin immunoprecipitation (ChIP) coupled to microarray analysis or ChIP-chip technology [45] that demonstrated a hierarchy of RE1 sites for preferential recruitment of REST. Canonical RE1 sequences show the strongest REST binding and control ubiquitous genes, whereas atypical motifs display weaker interactions, mainly involved in the regulation of tissue-specific targets [46,47].

We used this DN-REST construct to demonstrate that inappropriate expression of REST is responsible for an overall repression of the secretory pathway in a neuroendocrine variant cell line. Isolated from the rat pheochromocytoma PC12 line, the variant A35C lacks expression of markers of neurone-specific organelles, *i.e.*, synaptic vesicles and dense core granules. These proteins are missing at the mRNA level indicating a transcriptional block that is reversed by introduction of the DN-REST [31]. The concept of master switches emerged, transcription factors that can control expression of whole pathways or phenotypes by regulating expression of large subsets of genes.

### 3.3. Pasting Domains

Many transcriptional responses are very fast and transient, often as a result of the quick activation and degradation of the transcription factors involved. Some transcription factors are small or processed and, therefore, difficult to follow and visualise by conventional biochemical methods. A powerful approach to overcome these hurdles is the use of chimeric transcription factors, such as GAL4-VP16, that contain the DNA-binding domain of the yeast GAL4 transcription factor coupled to the activating domain of the herpes simplex VP16 protein [48]. The UAS-lacZ gene contains four copies of the UAS binding site for GAL4, following the same principle of the κb reporter described earlier, and is used to measure transcriptional activity.

The GAL4 system was used to study the nuclear localisation and transcriptional activity of elusive factors, such as Notch, a member of a transmembrane receptor family that transduce intercellular signals controlling cell fate [49]. The function of Notch was unclear, because examination of the intracellular domain of the single span membrane protein did not reveal any recognisable catalytic motif. With the aim of understanding how Notch signalling works, the GAl4-VP16 sequence was inserted in the protein, and the UAS-*lacZ* was introduced to serve as an indicator of activity. When GAL4-VP16 was inserted in the intracellular domain (ICD), nuclear localisation *in vivo* could be detected as activation of the UAS-*LacZ* transcription, whereas if inserted in the extracellular domain, access to the nucleus could not be shown. These experiments demonstrated that in response to extracellular signalling, Notch is cleaved and the ICD translocated to the nucleus. Though the presence of VP16 in these constructs increased the observed transcriptional activity, if GAL4 was inserted alone in the Notch ICD, transcriptional response was also detected, proving that Notch transduces extracellular signals by regulating expression of specific genes [50]. Using these constructs, it has been determined that Notch is cleaved on the plasma membrane by γ-secretase, a membrane protease implicated in the proteolytic cleavage of other substrates, such as β-APP, and, thus, implicated in Alzheimer’s disease [51]. Indeed, using a myc-his-tagged 57 amino acid *C*-terminal fragment of APP and a luciferase reporter gene with the EGFR promoter, it was confirmed that γ-secretase cleavage of various targets results in the translocation of ICDs to the nucleus to regulate gene expression [52].

The chimeric constructs combining DNA-binding domains with transcription-activating domains have been used for a wide range of studies, including the most fundamental transcription machinery. Thus the yeast *HIS4* core promoter was coupled with a Gal4-binding site 55 bp upstream of the TATA box, and this template was linked to streptavidin-coated magnetic beads via 5′-biotin. The beads were incubated with nuclear extracts from yeast transfected or not with Gal4-VP16, and the complexes formed were recovered by endonuclease cleavage. The components of the complexes were then analysed by quantitative mass spectrometry and Western blotting [53]. A comparison with a control promoter-less template identified the binding of the basal components of PIC, such as TFIIH, TFIID and the mediator. The presence of an activator (VP16) seemed to recruit mainly chromatin modelling proteins, such as the histone H3 acetyltransferase, SAGA, the histone H4 acetyltransferase, NuA4, and the ATP-dependent chromatin remodeller, Swi/Snf. The use of a different activator (Gcn4) revealed that though both recruit the same proteins, the levels of the recruited complexes are different, indicating that the particular arrangement of binding elements of individual promoters leads to multiple modes of promoter recognitions and, possibly, multiple types of PIC.

## 4. Live Reporters

The advent of reporter genes offered possibilities of studying transcriptional regulation *in vivo*. Transgenic mice have been generated introducing markers, such as β-gal, GFP or other fluorescent probes into the genome, downstream of the desired gene. Expression of the probe is regulated by the promoter of the studied gene, providing a marker to follow expression of genes throughout development and the different tissues and organs of the body [54,55].

A different strategy of *in vivo* genetic manipulation uses mobile DNA elements or transposons that “jump” through the genome. Though no function has been found for these elements, it was observed that, on occasion, insertions occur in regulatory regions changing gene expression. Transposons can also integrate in coding regions, therefore altering expression or even silencing the disrupted gene or causing mutations when exiting an integration site that is not repaired properly. These elements and the transposases involved in their movement have been developed into a widely used strategy to study and discover genes implicated in disease [56]. The engineering of PiggyBac-transposase mice to allow genome-wide mobilisation of the PiggyBac transposon induced a range of cancers, which when screened, revealed many cancer-associated genes. One such gene is Spic, which encodes a PU.1-related transcription factor that controls the development of macrophages and is associated to haematopoietic tumours [57].

The transposon technology has been applied to a variety of systems, including the study of genetic expression in *Plasmodium falciparum*, the main parasite causing malaria in humans and responsible for the more serious clinical cases of the disease [58]. The particularity of this parasite is that regulation of gene expression throughout its complex lifecycle is highly coordinated, particularly in the blood stages, when the parasite invades and multiplies in erythrocytes, causing the main clinical manifestations of the disease. A medium-scale PiggyBac transposon genome-wide mutagenesis screen of *Plasmodium falciparum* led to the identification of CAF-1 (ccr4-not associated factor 1), a component of the carbon carbolite repressor protein 4 (CCR4) that causes severe attenuation of intraerythrocytic growth *in vitro*. Functional characterisation of the CAF-1 null mutant revealed that it regulates mRNA decay. Using microarray analysis, we showed that CAF-1 has a critical role in temporal gene regulation, as its disruption delays overall gene expression, eventually inducing premature egress from red blood cells, thus attenuating the growth rate [59]. Transgenes enable the control of gene expression and follow-up *in vivo*, while the transposon technology is a forward genetics strategy that provides means to identify novel genes and understand their functions and associated phenotypes.

## 5. Concluding Remarks

**Figure 3:**
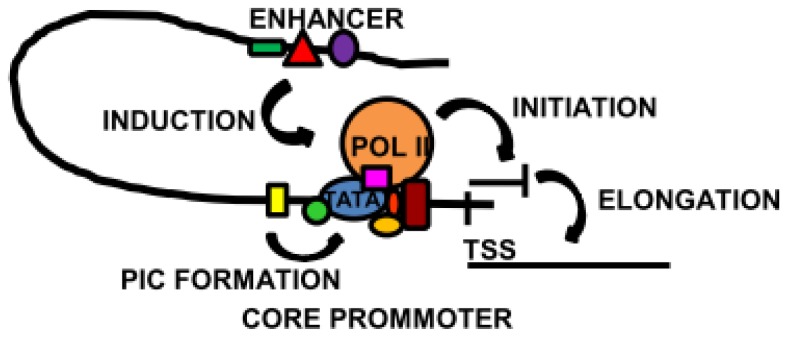
Overview of some of the main transcriptional mechanisms. The core promoter, localised around the transcription start site (TSS), contains binding sites for the basal transcription machinery. Specialised core transcription factors located close to the TATA box induce and stabilise pre-initiation complex (PIC) formation. In some cases, the core transcription factors promote the binding of Pol II and the initiation of transcription. Activation of a distal enhancer brings it into proximity of the core promoter, triggering transcription or elongation to synthesise full length mRNA.

The capability to manipulate regulatory sequences and accurately probe their activity led to the understanding of many regulatory mechanisms of gene expression (Figure 3). Thus, the regulatory regions in the flanking sequences of genes could be identified and dissected to characterise the transcription factors involved and their precise role in the regulation of particular genes. The importance of positioning on the DNA was recognised, raising the concept of enhancers and core promoters. Interactions between DNA-binding proteins to remodel chromatin, form the PIC, recruit Pol II and trigger transcription were identified. Detailed analysis of transcription factors was achieved by making constructs of modified proteins, revealing DNA-binding domains, activating domains, interactions with other transcription factors, as well as with the chromatin remodelling machinery that gave an insight into how these factors function. These tools progressed the understanding of gene expression in cells and organisms, establishing tissue specificity and developmental regulation.
